# Group Nature-Based Mindfulness Interventions: Nature-Based Mindfulness Training for College Students with Anxiety

**DOI:** 10.3390/ijerph20021451

**Published:** 2023-01-13

**Authors:** Luke A. Vitagliano, Kelly L. Wester, Connie T. Jones, David L. Wyrick, Amber L. Vermeesch

**Affiliations:** 1 Department of Counseling and Educational Development, University of North Carolina Greensboro, 1400 Spring Garden Street, Greensboro, NC 27412, USA; 2 Department of Public Health Education, University of North Carolina Greensboro, 1400 Spring Garden Street, Greensboro, NC 27412, USA; 3 Department of Family and Community Nursing, School of Nursing, University of North Carolina Greensboro, 1007 Walker Avenue, Greensboro, NC 27402, USA

**Keywords:** anxiety, college students, COVID-19, mental health, mindfulness, nature, attention restoration theory (ART), Nature-Based Mindfulness Training © (NBMT)

## Abstract

The mental health crisis across college campuses is accelerating, with anxiety listed as the top mental health issue for undergraduate college students. Although evidence suggests the COVID-19 pandemic escalated the mental health crisis on college campuses, pre-COVID-19 anxiety among college students was on the rise. Research supports Mindfulness Based Interventions (MBIs) to reduce anxiety among college students. Additionally, exposure to natural environments, which are accessible to students on college campuses, is effective in reducing anxiety. While brief nature-based mindfulness interventions appear effective in reducing anxiety among college students, these interventions are often offered in isolation without social interaction among group members and lack intentional integration of mindfulness and nature-related theories. The purpose of this work is to describe a framework for integrating the use of Mindfulness and Attention Restoration Theory (ART) in an innovative psychoeducational group intervention, Nature-Based Mindfulness Training © (NBMT), for college students with anxiety. In conclusion, we argue for the need to intentionally integrate mindfulness and nature into nature-based mindfulness interventions as an effective and sustainable means to reduce anxiety. Limitations and areas for future research are described.

## 1. Introduction

The mental health crisis across college campuses is accelerating, with anxiety listed as the top mental health concern among college students [1]. The World Health Organization estimates the COVID-19 pandemic triggered a 25% increase in the prevalence of anxiety and depression disorders worldwide [2]. College-aged students were particularly susceptible to increased anxiety symptoms during the COVID-19 pandemic [3], which sent universities into disarray, negatively impacting college students who experienced increased perceived stress [4], with upwards of 44% of students experiencing anxiety [5,6]. Although the COVID-19 pandemic expedited the mental health crisis on college campuses, college student anxiety was on the rise prior to the pandemic [7]. The number of students pursuing campus mental health resources has increased over the years [7,8]; however, college students may underutilize mental health services due to lack of information on campus resources, limited time due to busy schedules, and the stigma associated with disclosing mental health issues [9,10]. With the gradual return of campus life, college counseling centers, often under-resourced [11], must adapt to serve their communities, ripe with mental health concerns. The purpose of this work is to describe a framework for integrating the use of mindfulness [12] and attention restoration theory (ART) [13] in an innovative psychoeducational group intervention, Nature-Based Mindfulness Training © (NBMT), for college students with anxiety.

### 1.1. Background

Group counseling interventions can reach an increased number of students seeking services on college campuses, and groups such as mindfulness-based interventions (MBIs) have been found to reduce anxiety among college students [14]. However, group counseling services were impacted by the COVID-19 pandemic. Forced to leave campus and refrain from social gatherings, students were isolated and experienced increased levels of loneliness [15]. With COVID-19 still prevalent and students returning to campus, some may not want to gather indoors in group settings. One solution to refraining from indoor social gatherings is to connect outdoors, in nature.

Exposure to natural environments is effective in reducing anxiety [16,17,18,19,20] and improving mental health and well-being [21,22]. College students benefit from spending time in nature [23,24], with natural environments and greenspaces easily accessible on campuses [25]. Because both exposure to nature and mindfulness practices are effective in reducing symptoms of anxiety, engaging in mindfulness practice in nature may have a greater impact on anxiety and enhance well-being [26,27]. Nature-based mindfulness connects nature with mindfulness techniques, with recent explorations supporting the benefits of nature-based mindfulness interventions for college students [28,29,30,31,32].

#### 1.1.1. Mindfulness

Kabat-Zinn’s definition of mindfulness, “the awareness that emerges through paying attention, on purpose, in the present moment, nonjudgmentally to the unfolding of experience, moment by moment” [33] (p. 4), is the most widely accepted, even though other definitions of mindfulness have been proposed [34,35,36]. His definition informs a model of mindfulness that includes three bidirectional axioms: *Attention*, “paying attention”; *Attitude* “in a particular way”; and *Intention* “on purpose”; which interact simultaneously in a moment-to-moment process [12] (p. 375). Attention, or the “what,” is paying attention, moment to moment, to both internal and external experiences. Individuals can pay attention to their heartbeat (i.e., internal), or what they see (i.e., external) to increase awareness of their experience in the moment. The second axiom, Attitude, “how” one attends to the present moment, is integral to the interpretation of experience. Attention to the moment with a poor attitude may lead one to experience negative affect and be judgmental or self-critical, while an open mind may lead to positive affect, kindness, and acceptance. The final axiom, Intention, is one’s purpose or goal for mindfulness, otherwise known as the “why.” An individual who is aware of their inability to self-regulate, when experiencing negative affect or distorted thinking, can identify self-regulation as a goal to guide their actions for mindfulness practice. One’s Intention often changes and evolves based on the interaction and interpretation of the present moment, ranging from self-regulation to self-liberation [12]. When combined, Attention, Attitude, and Intention facilitate a shift in perspective towards objectivity, coined *Reperceiving*, the meta-mechanism of mindfulness (i.e., theory of change).

By increasing the ability to be objective to internal and external experiences, Reperceiving can lead to additional mechanisms such as (1) self-regulation of emotions, (2) exposure (i.e., distress tolerance), (3) clarification of values, and (4) cognitive, behavioral, and emotional flexibility, in turn, contributing to positive health outcomes [12]. Therefore, mindfulness is a process, with the simultaneous interactions of the three axioms leading to Reperceiving, tapping into additional mechanisms to influence mental health outcomes (e.g., mindfulness → reperceiving → additional mechanisms → mental health outcomes). Mindfulness is inherently complex, leading researchers to examine the multi-faceted construct of mindfulness [37] and its impact on psychological outcomes [38].

#### 1.1.2. Measuring Mindfulness

In dissecting the complex structure of mindfulness, five facets of mindfulness were identified and developed into the Five Facet Mindfulness Questionnaire (FFMQ) [37] and are connected to the previously described model of mindfulness [12] (Table 1). The first facet of mindfulness, *Observing*, involves noticing internal and external experiences (i.e., thoughts, emotions, sensations), while the second facet, *Describing*, involves using words to label one’s internal and external experience [39]. Both Observing and Describing capture the Attention axiom of mindfulness. The third facet of mindfulness, *Acting with awareness*, involves the ability to attend and intentionally act in the moment [39] and is in line with the Intention axiom of mindfulness. The fourth facet of mindfulness, *Nonjudging of inner experience*, refers to a neutral and objective stance towards feelings and thoughts, while the fifth and final facet, *Nonreactivity to inner experience,* involves the ability to allow thoughts and feelings to come and go without becoming involved with them [39]. Both Nonjudging of inner experience and Nonreactivity to inner experience capture the Attitude axiom of mindfulness. See Table 1. Connection of the Five Facets of Mindfulness to the Model of Mindfulness Axioms for further detail.

A common theme across studies exploring the individual five facets of mindfulness is the nonsignificant relationship, or at times, positive relationship between Observing and psychological adjustment. The Observing facet frequently has no relationship with psychological symptoms [37,39,40,41,42]; however, a few researchers have found positive relationships between Observing and stress [38] and anxious arousal in a clinical sample [43]. Observing involves noticing internal and external experience, foundational to mindfulness practice, yet higher Observing is associated with increased judgment [38] and maladaptive responses to increased awareness [43]. Individuals with little to no meditation experience may observe in a way that is maladaptive and not consistent with other integral aspects of mindfulness (i.e., Nonjudging of inner experience, Nonreactivity to inner experience, Acting with awareness) [41,44]. It is how one observes their experience [45] and the ability to act with awareness [46] that are integral in managing anxiety. Additionally, with Attention integral to mindfulness practice, Describing is correlated with a decrease in anxiety-related symptoms [46].

#### 1.1.3. Mindfulness Based Interventions

Evidence supports MBIs decreasing suicidal ideation and depression [47], stress and anxiety [48], social anxiety [49], and test anxiety [50]; and improving satisfaction with life, mindfulness, and self-compassion [51] among college students. A theme across structured MBIs such as Mindfulness Based Stress Reduction (MBSR), Mindfulness Based Cognitive Therapy (MCBT), and Dialectical Behavior Therapy (DBT) is the duration of the intervention (i.e., at least 8 eight weeks) and frequency of meetings (i.e., at least one time per week). MBIs with eight or more sessions produced greater effect sizes than interventions with less than eight sessions [14]. However, given college students busy schedules [52], the time required to participate in structured MBIs may be inaccessible, and therefore MBI may be unable to engage students experiencing anxiety [29]. College students prefer MBIs that are brief, delivered face-to-face, and can be easily incorporated into their busy schedules, whether brief in nature or through course curriculum [53]. Not only do universities have MBIs to support college students with anxiety, but campuses also have another resource to improve mental health, access to nature [25].

### 1.2. Nature

The belief in the benefits of exposure to nature in human health spans thousands of years, from Aristotle [54] to more recent transcendentalists such as Ralph Waldo Emerson [55] and Henry David Thoreau [56], and naturalists like John Muir [57]. Exposure to nature is linked to positive mental health benefits such as reducing anxiety [18,20,58] and enhancing well-being [59]. Nature has been defined in a variety of ways [60,61,62] leading to several theoretical models explaining the impact of the influence of nature in the human–nature connection [13,63,64,65]. ART is one of the dominant nature-based theories suggesting natural environments are the antidote to recover from mental fatigue by restoring attentional capacity [13,66].

#### 1.2.1. Attention Restoration Theory

Derived from the work of William James [67] the founders of ART posit mental fatigue arises with prolonged use of *directed attention*, forced attention that requires a great deal of effort on something that is not particularly interesting, while *involuntary attention* requires no effort at all and has the capacity to restore mental fatigue [13]. The authors propose four components found in natural environments to provide the opportunity to rest directed attention, thus restoring cognitive capacity and reducing mental fatigue. The first component, *Being Away*, entails a physical distancing, or escape, of oneself from their current day-to-day environment. Kaplan and Kaplan [13] identify three patterns from escape: distraction, putting aside ordinary work, and an internal escape (i.e., escape from mental efforts, taking a rest from pursuing current purposes). They suggest the strongest effect is through a combination of these patterns. The second component, *Extent*, includes the scope and sense of relatedness one experiences in natural environments. Scope suggests there is more beyond what is immediately perceived, while relatedness refers to the perceptions that the elements of a setting are part of a larger whole, with Extent prompting a sense of fascination in the natural world. The third component, *Soft Fascination*, occurs when one is engaged in involuntary attention induced by the natural aesthetics of the environment (e.g., trees, clouds, sunsets, rivers), which captures attention, but does not require directed attention, restoring focus, concentration, or mental capacity, thus reducing fatigue [13,66]. The fourth component, *Compatibility*, suggests natural environments are well suited to one’s preference or purpose within that setting [66]. Therefore, the four components in natural environments (i.e., Being Away, Extent, Soft Fascination, and Compatibility), induce involuntary attention, which restores mental fatigue (e.g., nature exposure → involuntary attention → restore mental and cognitive capacity → positive health outcomes).

#### 1.2.2. Nature Access and Anxiety

Spending time in nature is linked to positive mental health outcomes including reducing anxiety [16,17,18,19,20]. Higher percentages of green space around one’s home is correlated with reducing the impacts of stressful life events [68] and lower levels of depression, stress, and anxiety [69]. Although the research evidence supports the mental health benefits from exposure to nature, individuals may have little to no access to nature.

The push towards urbanization and the increase in access to technology leads human beings to spend more time indoors, decreasing regular contact with nature, and may explain the increase in mental illness [70,71]. When access to nature is limited, nature-based guided imagery [72], natural sounds [73], and virtual reality psychotherapy [74] aid in reducing anxiety. While technological advances aid in reproducing the benefits of nature exposure, scholars suggest conservation efforts to preserve botanical gardens [75] and local parks [59,76] as they are integral to promoting human health. Natural environments are easily accessible to college students, with evidence supporting nature exposure to be beneficial for college student mental health.

#### 1.2.3. Nature Exposure and College Students

College students use campus greenspaces regularly and consider them essential to the campus environment [77] to alleviate stress [78] and improve perceptions of quality of life [24]. Greenspaces on college campuses are therapeutic as they enhance the physical, mental, and social well-being of students [23]. Students can engage with nature actively (i.e., exercise) or passively (i.e., sitting, studying), with as little as 5 to 10 min of active nature exposure [79] and 10 to 20 min of passive nature exposure [80] improving mental health.

In the 21st century, institutions of higher education have started to promote, develop, and preserve natural environments on college campuses with evidence supporting mental and physical health benefits for college students [25,81]. With the alarming rates of anxiety among college students, institutions of higher education must prioritize developing and maintaining greenspaces for students to access regularly on campuses. Not only is accessibility to nature important, but also the ways in which one engages with natural environments [82]. Collectively, institutions of higher education, college counseling centers, researchers, and practitioners may be able to maximize mental health benefits for students by developing nature-based health interventions (NBIs) designed to increase nature exposure and reduce mental health symptoms.

### 1.3. Nature-Based Health Interventions

Policymakers and healthcare professionals have increasingly advocated for NBIs to promote improved health and well-being amid the demand for mental health services [83,84]. NBIs are strategies, activities, and programs designed to engage people in nature-based experiences aimed to improve physical, mental, and social health and well-being [85]. Nineteen experts across the globe participated in a Delphi study to identify two broad categories of NBIs, those focused on changing the environment (i.e., increased provision of public urban parks and gardens, indoor plants) or changing human behavior (i.e., ecotherapy, green exercise) [85]. NBIs can be a cost-effective solution to improve mental health and well-being [28,85] across a variety of domains (e.g., physiological, psychological, social) [86], with NBIs focused on changing human behavior effective in reducing anxiety [58,84,87].

A variety of factors must be considered when selecting, developing, and implementing NBIs to include financial cost, anticipated health benefits, accessibility, and the capability to deliver the intervention [85,88]. When implementing interventions with college students, the engagement and attractiveness of the intervention must be considered [88]. Students prefer interventions that are brief and can be easily integrated into their busy schedules [53]. Given that college students have access to nature, and both NBIs and MBIs reduce anxiety, scholars suggest connecting with nature enhances mindfulness practice and well-being [26,27].

### 1.4. Nature-Based Mindfulness

Nature-based mindfulness connects nature with mindfulness techniques to deepen meditation practices [27], encourage social activism, assist in coping with climate change [89], and improve well-being [90,91]. Mindfulness is an integral component of Shinrin-Yoku (i.e., forest bathing) [92], which aids in reducing anxiety [93]. Although the practice of integrating nature and mindfulness has been used for centuries [26], the evidence in support of nature-based mindfulness interventions to improve human health and well-being is in its infancy.

#### 1.4.1. Nature-Based Mindfulness Interventions

Recently, nature-based mindfulness interventions have been piloted in adventure therapy and outdoor education programs to study their impacts. Young adult males with substance use disorders who engaged in mindfulness-based experiences during a 90-day adventure therapy program increased scores in Nonjudging of inner experience and Nonreactivity to inner experience from the FFMQ, which correlated with a decrease in overall subjective distress [94]. Fourteen adults participating in an 8-day Outward Bound Mindfulness Program, with five hours per day dedicated to mindfulness practice experienced a significant increase in mindfulness and positive affect scores post-intervention and maintained these levels at 3-month follow-up when compared to a control group [90]. While evidence from these pilot studies supports the positive benefits of nature-based mindfulness interventions in outdoor education and adventure therapy programs, these programs may be inaccessible due to the financial cost [95] and time required (8 days to 90 days) to be in a remote natural environment.

#### 1.4.2. Nature-Based Mindfulness Interventions and College Students

Danish university students experiencing moderate to high levels of stress who participated in a 5-day residential mindfulness retreat aimed at reducing stress and improving mental health were randomly assigned to one of three conditions: indoors, natural outdoor setting, and a control group [28]. Contrary to previous research from a systemic review and meta-analysis on the effects of mindfulness training in outdoor settings [96], the authors did not find a difference in mindfulness and perceived stress scores for participants in the indoor and natural outdoor settings [28]. Although researchers found a moderate effect size post-treatment for perceived levels of stress between the indoor and control group participants, the results were nonsignificant. While they attribute this finding to the design of the indoor environment emulating the restorative qualities of natural environments [28], given the small sample size (*n* = 60) and observed power not reported, the results may be subject to a type II error. While an advantage of this program is the low attrition rate (3%), college students prefer mindfulness interventions with brief time commitments [53]. Brief nature-based mindfulness interventions may provide positive mental health benefits and are more accessible than those offered in remote natural settings and residential programs.

Few researchers have explored the mental health impacts of brief nature-based mindfulness interventions among college students. Researchers at a mid-Atlantic university piloted a 1-min and 5-min nature-based mindfulness walk with participants asked to respond to written comment cards at the end of each walk to assess the psychological effects of the intervention [30]. Although 96% of the participants indicated positive psychological effects, with 82% indicating stress reduction as the most common effect, only 60% of participants completed the written comment cards. While the results are promising, this study poses several limitations, which include no comparison or control group, no use of standardized assessments, and no pre-test scores. In exploring the impact of interventions on college student mood disturbance, students were randomly assigned to either the outdoor or indoor condition, and in each location were assigned to participate in a 20-min meditation or a control condition, writing daily activities in a typical week [29]. Students in the outdoor condition experienced a greater reduction in mood disturbance compared to the indoor condition regardless of activity, and students participating in the 20-min meditation experienced a greater reduction in mood disturbance compared with the control task, regardless of location. Although there was no added benefit of meditation in the outdoor location in this study, evidence supports brief meditation in nature enhances mental health benefits for college students compared to meditation indoors, and a control group [31]. Similarly, in exploring the impact of a 20-min walk on mood, students walking outdoors with and without mindfulness experienced an increase in positive mood compared to those walking indoors, while students walking outdoors with mindfulness experienced a greater reduction in negative affect compared to the other two conditions [32]. While the preliminary evidence supports mental health benefits of brief nature-based mindfulness interventions, students engaged in these interventions without social interaction. Social prescribing is correlated with improvements in social connectedness, physical health, and mental well-being [97]. While there is limited evidence regarding social interaction among participants in brief nature-based mindfulness interventions, enhancing social connectedness and allowing group members to learn from one another may enhance the benefits of brief nature-based mindfulness interventions.

The brief nature-based mindfulness interventions reviewed lack details of the integration of nature and mindfulness in the intervention design. We argue the need to intentionally integrate theoretical components of nature and mindfulness into nature-based mindfulness interventions given the reciprocal relationship between mindfulness and nature [27]. With both exposure to nature and mindfulness practice suggested to restore attention [98], ART may be suitable to integrate with mindfulness in nature-based mindfulness interventions aimed at reducing anxiety as it describes a process as to how nature positively impacts human beings.

## 2. Integrating Mindfulness and Attention Restoration Theory

**Figure 1:**
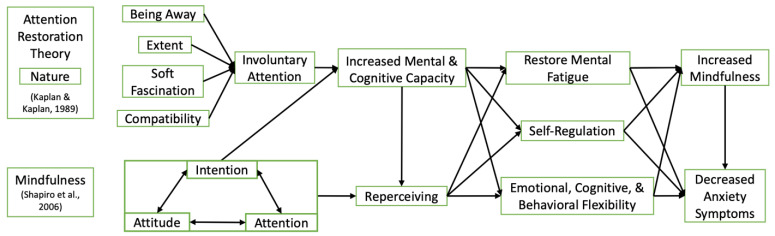
Integrating Core Components of Attention Restoration Theory and Mindfulness [12,13].

When integrating nature exposure into the therapeutic process, it is vital to identify the proposed causal pathways (i.e., theory of change) specific to the type of nature experience, the specific intervention components, and the intended mental health outcomes [99,100]. Mindfulness enhances the restorative characteristics of an environment, and natural environments aid in the development of mindfulness practice; both are suggested to restore attentional capacities, thus restoring mental fatigue [98]. ART suggests involuntary attention induced by natural environments leads to increased cognitive capacity to restore mental fatigue, while mindfulness suggests intentional attention to the present moment leads to a shift in perspective. With both nature activities and quiet activities compatible with reflection [101], once involuntary attention is induced by nature, the pleasing aesthetics of natural environments offset the potential discomfort of mindfulness practice, expediting Reperceiving. We propose the integration of the core components of mindfulness (i.e., Attention, Intention, Attitude, and Reperceiving) [12] and ART (i.e., Being Away, Extent, Soft Fascination, and Compatibility) [13]. As indicated in Figure 1, the core components of mindfulness are integrated once involuntary attention has been stimulated, informing the design of NBMT ©. Mindful engagement in nature can be used to refocus attention, respond with less reactivity, and enhance clarity of one’s internal and external experiences. Reperceiving partially mediates the relationship between connection with nature and mindfulness, specifically the Nonreactivity and Observing facets of mindfulness, suggesting that those who are more observant and less reactive may be less inclined to over-identify with thoughts and emotions [102]. Deidentifying with one’s subjective experiences (i.e., Reperceiving) to be more present in and connected to nature may restore mental fatigue, improve self-regulation, and enhance emotional flexibility, contributing to improved psychological symptoms (i.e., anxiety) [103]. See Figure 1. Integrating Core Components of Attention Restoration Theory and Mindfulness for further detail.

### 2.1. Nature-Based Mindfulness Training

NBMT © is a brief psychoeducational group intervention designed to be delivered outdoors, in nature, with the intended outcomes to improve mindfulness and reduce anxiety. NBMT © integrates theoretical constructs of Mindfulness [12] and ART [13] and includes psychoeducational content, experiential activities, and interaction among group members. The content and activities are designed to teach participants tangible skills, which are practiced during NBMT ©, that can be utilized after the conclusion of the intervention. Group discussion fosters a sense of community and social connectedness allowing participants to realize they are not alone in their struggles with anxiety and share their experiences in a safe place to learn from one another [104].

#### 2.1.1. Intervention Location

While college campuses provide access to natural environments [25], NBMT © is flexible and can be implemented with several and diverse populations and in a variety of locations. The location for the NBMT © intervention must:

1. Be in a natural environment;
2. Be easily accessible to participants;
3. Have limited external stimulus (i.e., foot traffic, car traffic);
4. Consist of an alternative location for inclement weather (i.e., open air structure, canopy).

The natural environment selected is recommended to be within walking distance or a short drive to participants, such as local parks, botanical gardens, and greenspaces, to increase accessibility to participants. Additionally, NBMT © is appropriate for those with physical limitations as the location must be accessible to those with physical disabilities. Participants are seated on the ground in a circle to be able to see everyone in the group. As participants are seated for the majority of NBMT ©, comfortable seating (i.e., blankets, camp chairs) can be provided for the duration of the intervention.

#### 2.1.2. NBMT © Journal

Participants are provided a paper journal and writing instrument to use throughout NBMT ©. The journal is 5.5′′ × 8.5′′ and consists of 16 pages and follows the sequence of the NBMT © curriculum. The psychoeducational content in NBMT © is packaged in acronyms, which are easy to remember, while the prompts for experiential activities provide space in the journal for participants to write or draw their responses. Rather than solely focusing on the Observing facet of mindfulness, the intentional use of a journal in NBMT © targets the Describing facet, which is correlated with a decrease in anxiety symptoms [46]. Additionally, during the intervention, the journal cannot be accessed on cell phones or electronic devices to allow participants to get away from both their typical day-to-day lives and technological distractions. At the end of NBMT ©, participants keep their journals with the psychoeducational content and experiential activities easily accessible at the conclusion of the intervention.

#### 2.1.3. Duration and Participants

NBMT © is 90-min in length and consists of eight to twelve group members. As NBMT © is specifically designed to decrease anxiety, participants must experience clinically significant distress due to their anxiety symptoms. Both the number of participants and length of NBMT © are consistent with recommendations for psychoeducational groups [104,105], including group MBIs such as dialectical behavior therapy (DBT) [106] and are more accessible than multi-day or remote nature-based interventions.

#### 2.1.4. Group Facilitator Characteristics

NBMT © group facilitators believe that natural environments can heal, promote mental health and wellness, and influence the therapeutic process. Throughout NBMT ©, the group facilitator follows a structured guide providing psychoeducational content, directs experiential activities, and facilitates discussion among participants. While the guide provides a format for the group process, group facilitators are encouraged to be flexible when using group counseling skills to focus, facilitate, and enhance group discussion to foster a safe and trusting environment and allow group members to learn from each other. Authenticity of the group facilitator is vital, with appropriate self-disclosure from the facilitator offering hope to participants by sharing their process of managing anxiety through nature-based mindfulness practices. Additionally, group facilitators must be attuned to the individual group participants and group dynamics and be flexible to effectively manage group dynamics.

### 2.2. Application of Nature-Based Mindfulness Training

Behavioral interventions can be considered as a combination of a set of intervention components [107], with each component highlighted in the application of NBMT ©. Table 2 provides the time allotted, a brief description of the individual intervention components, and the core components of Mindfulness and ART identified in Figure 1. The group facilitator introduces themselves and asks participants to introduce themselves to the group (i.e., Group Introduction). The facilitator serves as a model for participants, creating safety and trust within the group, a primary strategy of practitioners providing nature-based therapies [108]. After Group Introductions, the facilitator draws attention to the natural environment with Nature Restores by highlighting the setting of the group in nature (e.g., green space, park, arboretum, etc.) incorporating core components from ART (e.g., Being Away, Extent, Soft Fascination, Compatibility). The facilitator guides the discussion of participants’ experiences with nature and emphasizes the benefits of nature exposure in reducing anxiety. See Table 2. NBMT © Intervention Components: Time and Description for further detail.

After Nature Restores, the facilitator directs participants to label their anxiety in a reflective drawing and writing activity (i.e., Anxiety). The phrase “name it to tame it” coined by Dr. Daniel Siegal suggests naming an emotion increases the ability to tame or manage this emotion [109]. The Anxiety component incorporates affective labeling of emotions, a form of implicit emotion regulation [110] effective in reducing pre-competition anxiety in athletes [111] and reducing public speaking anxiety [112]. In naming their anxiety, the facilitator guides participants to set their intention for NBMT ©, to reflect on their purpose (i.e., the why) for participating, and to write their responses in the journal (i.e., Why NBMT ©?). After setting intentions for NBMT ©, the facilitator encourages participants to reflect on larger life goals and what life would look like without anxiety (i.e., Life Goals). Participants are directed to draw and write this goal to share with the group. With Intention a core component of mindfulness [12] found in MBIs [113], participants identify intentions both for their time during NBMT © (i.e., Why NBMT ©?) and a more global intention in their life (i.e., Life Goals).

Following the discussion on Life Goals, the facilitator shifts the discussion to guide participants to pay attention to their internal experience (e.g., thoughts, emotions, physical sensations) in the present moment (i.e., What is NBMT ©?). Participants are directed to write down their present thoughts, physical sensations, and emotions, and to share with the group which of these was easiest and hardest to recognize. The group facilitator highlights the importance to focus first on which is easiest to recognize (i.e., thoughts, emotions, physical sensations) within each participant to better recognize symptoms of anxiety. Participants take a short stretch break and sit in a different location prior to the facilitator directing participants to focus on their external experience. The stretch break marks the halfway point providing participants a different view of the natural environment and allows the facilitator a more direct view of participants who were sitting directly next to the facilitator at the start of NBMT ©. The facilitator guides participants to focus on their external experience, specifically the environment, surroundings, and the natural world asking participants to share with the group what they notice within their environment (i.e., Nature-Based Grounding). The facilitator directs participants to pay attention to their senses and participate in a grounding exercise, write responses in their journal, and share what they noticed in their external environment.

After the grounding exercise, the facilitator provides information on How to Practice NBMT © highlighting the importance of being open to new experiences and honest with themselves, to focus on one thing at a time, without judgment. In learning the core components of NBMT ©, the facilitator will prepare clients to participate in a guided meditation. The facilitator asks participants to find a comfortable position, provides instructions for breathing, and directs participants to engage with the meditation openly and without judgment.

The group facilitator guides participants through a 10-min Nature-Based Guided Meditation specific to the location of the NBMT © intervention site. The meditation first focuses on How to Practice NBMT ©, followed by What is NBMT ©?, with participants directed to pay attention to their internal experience. The meditation shifts to Nature-Based Grounding, with the facilitator guiding participants to focus on their senses to attend to their external experience (e.g., the natural environment). The end of the meditation focuses on intentions for NBMT © and broader life goals (i.e., Why NBMT ©?, Life Goals), specifically focusing on the core component from ART, Compatibility, highlighting reciprocity between intentions and nature. Following the Nature-Based Guided Meditation, the facilitator directs participants to reflect on three prompts and write their responses in the journal (i.e., Reflective Journaling).

Both Reflective Journaling and Group Conclusion directs participants to reflect on their experience and write responses to enhance or expedite Reperceiving. Reflective Journaling is incorporated towards the end of NBMT ©, with journaling effective in reducing anxiety symptoms among undergraduate students [114]. The three prompts focus participants on reflecting and writing responses to enhance the Attention, Attitude, and Intention components of Mindfulness [12]. NBMT © concludes with the participants writing their three takeaways from their time in the group. The facilitator will share their top takeaway and ask each participant to share their top takeaway from NBMT © with the group (i.e., Group Conclusion).

## 3. Discussion

NBMT © is an attractive, engaging, effective, sustainable, and cost-effective intervention designed to reach more college students seeking counseling services and reduce anxiety symptoms. Researchers developing a grounded theory identified four themes on how practitioners experience, perceive, and work with nature towards therapeutic goals [108]. Integrated in the NBMT © design, the first three themes include a belief that nature is actively influencing the therapeutic process, the practice of working with nature, and the relationship between the practitioner and nature. The fourth theme, creating conditions for clients’ engagement through nature, includes five primary methods of intervention all evident in the NBMT © curriculum:

1. Creating safety and trust;
2. Facilitating internal and external awareness;
3. Teaching new ways of knowing;
4. Role modeling and invitations;
5. Helping clients in meaning-making.

As an intervention strategy, the social interaction among participants is essential to enhance the benefits of NBMT ©. It is vital group facilitators create a safe environment for group members in the early stages of the intervention. Modeling authenticity through appropriate self-disclosure early in the intervention allows group members to experience the power of vulnerability, establishes safety, and sets the stage for others to share their experience to learn from one another.

The developer intentionally designed NBMT © to invoke involuntary attention, prior to introducing Why NBMT ©? approximately 20 min into the intervention, as evidence suggests 10 to 20 min of nature exposure in college students leads to mental health benefits [80]. Why NBMT ©? aims to increase awareness of group member intentions for practicing nature-based mindfulness and participating in NBMT ©, to create meaning out of their experience, and recognize mindfulness practice is compatible with nature. Focusing on intentions (i.e., Why NBMT ©? and Life Goals) fosters a sense of hope in participants that NBMT © and nature-based mindfulness practice will aid in reducing anxiety and support broader life goals prior to introducing content specific to Attention. What NBMT ©? and Nature-Based Grounding targets internal and external awareness, both integral in mindfulness practice [12] and therapeutic interventions in nature [108]. In DBT, Mindfulness “What” and “How” skills are effective in reducing anxiety among college students [50]. How to Practice NBMT ©? fosters new knowledge on how to practice mindfulness openly, honestly, and nonjudgmentally, vital to reducing symptoms of anxiety [45].

The Nature-Based Guided Meditation is an experiential activity to put learning into practice. While the majority of the Nature-Based Guided Meditation remains consistent, the group facilitator is tasked with developing the Nature-Based Grounding portion of the meditation script. This part of the script is designed to be flexible and incorporate specifics of the natural environment where NBMT © is conducted. The group facilitator is required to develop alternatives within the meditation script to highlight the current weather conditions and wildlife present during the NBMT © group (i.e., sun, wind, birds, etc.) and effectively guide participants through the elements of the natural environment in the present moment. Intentionally administering the Nature-Based Guided Meditation to include specific aspects of the present natural environment is vital for participants to respond to the Reflective Journaling prompts. Finally, during the Group Conclusion, the facilitator continues to model safety and trust, in self-disclosing their top takeaway from the group and inviting participants to share their takeaway to make meaning of their experience of NBMT ©. The social interaction among the group facilitator and members throughout NBMT © is intentionally designed as an innovative strategy to enhance the benefits of NBMT ©.

### 3.1. Social Interaction

While there is limited evidence highlighting social interaction among participants in brief nature-based mindfulness interventions, the importance of social interaction is foundational to Wilderness Therapy (WT). The Concurrent Model of the WT Process highlights the Social Self as one of three primary components inherent in the WT process [115], which is informed by Social Learning Theory (SLT). SLT posits people can learn a new behavior through observing others by attending, retaining, reproducing, and identifying motivating factors to reproduce the behavior [116]. In NBMT ©, the group facilitator models vulnerability, authenticity, and appropriate social interaction in the group setting, fostering a safe learning environment, to set the stage for members to share their experiences to learn and grow from each other. As an intervention strategy, NBMT © group facilitators are seen as approachable, relatable, and in a positive light, just as WT clinical staff are perceived more positively by clients than counselors in other settings [117]. These group facilitator characteristics allow for group members to learn from each other, increase self-efficacy, and improve mental health functioning. By translating these characteristics, NBMT © offers an innovative strategy to aid in the development of group nature-based interventions that are attractive, are more accessible than remote nature-based services, and can reach more clients than individual counseling.

### 3.2. Limitations

While there are strengths to integrating ART and Mindfulness in brief group nature-based mindfulness interventions there are limitations of the application of NBMT © to consider. First, the facilitator is unable to protect the confidentiality of participants in the outdoor setting, a necessary component for NBMT ©. Second, weather may be a limitation (e.g., temperature, precipitation), with additional preparations for inclement weather necessary (i.e., shifting the group to a covered outdoor structure). Third, for nature-based counseling interventions it may be challenging to determine the impact of nature versus the intervention itself on client outcomes [99,118]. Fourth, group counseling interventions may present challenges with managing individual group members and the group dynamics. Facilitators of NBMT © must possess group counseling competencies and feel comfortable to address challenges that arise in the group setting. Finally, NBMT © may not be ideal for all students struggling with anxiety, especially when more acute mental health concerns are present (e.g., suicidality, crisis) and individual counseling may be more appropriate.

### 3.3. Future Directions

NBMT © is designed to be simple and flexible, as these are top predictors for program adoption [119], with the ability to be adapted to each university, school, organization, and agency based on their clinical and environmental resources. Currently, the developer of NBMT © is in the process of conducting a pilot study to assess feasibility of the intervention and identify preliminary outcomes of anxiety and mindfulness scores among college students when compared to a control group. The research team will evaluate several distinctive features of feasibility to include recruitment and sample characteristics, procedures and measures, intervention acceptability, and the identification of resources needed to implement and manage the study, and evaluate participant responses and outcomes to NBMT © [120]. Once feasibility is established, the Multiphase Optimization Strategy (MOST) [105,121] can be utilized. MOST is a methodology intended to build, optimize, and evaluate behavioral interventions which are comprised of a set of intervention components. The research team hopes to employ MOST in the future to iteratively examine the impact of each intervention component on the desired outcomes and strengthen each component against an a priori performance standard to create an optimized version of NBMT © that can then be evaluated in a fully powered 2-arm randomized control trial. A brief screening tool can be utilized, such as the two-item Generalized Anxiety Disorder-2 (GAD-2) [122] to ensure participants are experiencing clinically significant distress due to their anxiety symptoms. Several group facilitators will be needed to further examine the impacts of NBMT ©, with a train-the-trainer model currently under development.

To provide NBMT © to college students, it is vital for group facilitators to develop and maintain relationships with institutions of higher education, college counseling centers, and various departments on campus. With college students’ busy schedules [52], facilitators may offer several group times for college students to sign-up or offer the NBMT © intervention through course curriculum [53]. While the interaction of group members is integral in NBMT ©, it is designed to be flexible with various potential applications of the intervention components.

In a world increasingly run by technology, researchers can adapt and utilize technology as a resource to improve mental health and wellness through e-health interventions. With evidence of nature-based guided imagery [72], natural sounds [73], and virtual reality psychotherapy [74] reducing anxiety, technological adaptations must be considered for NBMT ©. As NBMT © is comprised of a set of intervention components, the NBMT © mobile application may present participants with a booster after the group intervention to maintain treatment outcomes over time. Additionally, a mobile application can be implemented as a standalone e-health intervention by increasing the reach of the NBMT © curriculum to improve health and well-being. As the mobile application for NBMT © would be an e-heath intervention, the MOST methodology can be used to enhance and optimize the intervention [121].

Additionally, anxiety is not the only mental health diagnosis of concern among college students. Although NBMT © is designed to reduce anxiety, the intervention can be adapted specifically for other mental health concerns by replacing the Anxiety intervention component with a different mental health concern (i.e., depression), with nature-based mindfulness interventions reducing depressive symptoms among college students [31]. NBMT © can be implemented with other populations (i.e., high school students; high-stress professionals) experiencing mental health concerns. NBMT © may not only provide a creative and innovative solution to serve college students seeking support, but it may also fuel research and development of nature-based mindfulness interventions to support students and high-risk populations experiencing a variety of mental health concerns.

## 4. Conclusions

Institutions of higher education must maximize their campus resources and identify creative strategies to combat the mental health crisis on college campuses. While group MBIs and exposure to nature improves college student mental health, there is limited available evidence on the impact of nature-based mindfulness interventions for college students experiencing anxiety. Additionally, nature-based mindfulness interventions with college students lack the articulation of integrating nature and mindfulness and nature-related theories in the nature-based mindfulness intervention design. We argued the need to intentionally integrate theoretical components of nature and mindfulness into nature-based mindfulness interventions and provide an example of an innovative brief nature-based mindfulness intervention taking place in a group setting. In this manuscript, the core components of Mindfulness [12] and Attention Restoration Theory (ART) [13] were reviewed, integrated, and applied to college students experiencing anxiety through NBMT ©, an innovative psychoeducational group intervention aimed at reducing anxiety and improve mindfulness.

The authors highlight evidence of the benefits of mindfulness practice and interacting with nature in reducing anxiety-related symptoms, an epidemic across college campuses. With college counseling centers facing an influx of students experiencing anxiety, NBMT © offers an innovative and creative strategy to meet college student mental health needs. NBMT © integrates theoretical components from Mindfulness and ART as a psychoeducational group intervention that may benefit college students struggling with anxiety and improve mental health and well-being on college campuses.

## Figures and Tables

**Table 1:** Connection of the Five Facets of Mindfulness to the Model of Mindfulness Axioms.

Mindfulness Axioms	Attention	Intention	Attitude
Facets of Mindfulness	Observing Describing	Acting with awareness	Nonjudging of inner experience Nonreactivity to inner experience

**Table 2:** NBMT © Intervention Components: Time and Description.

Intervention Component	ART & Mindfulness Components	Time	Description
Group Introduction	Being Away Intention	0:00–0:05	Facilitator and participants share their name, pronouns (optional), major, and reason for participating in NBMT ©.
Nature Restores	Being Away Intention	0:05–0:10	Facilitator draws attention to natural environment and guide discussion with participants on their experiences with nature
Anxiety	Compatibility Attention	0:10–0:20	Facilitator directs participants to reflect on their anxiety and to write or draw how anxiety impacts their lives.
Why NBMT ©?	Compatibility Intention	0:20–0:25	Participants identify their purpose for participating in and practicing NBMT © and write their purpose.
Life Goals	Compatibility Intention	0:25–0:35	Participants reflect on their larger life goals, drawing or writing this goal and sharing with group members.
What is NBMT ©?	Soft Fascination Attention	0:35–0:45	Participants focus on their internal experience (i.e., thoughts, emotions, and sensations) and write what they notice in the moment.
Nature-Based Grounding	Extent Soft Fascination Attention	0:45–0:55	Participants focus on their external experience (i.e., surroundings, natural environment) and facilitator guides participants in Nature-Based Grounding exercise.
How to Practice NBMT ©?	Being Away Compatibility Attitude	0:55–1:05	Facilitator guides discussion among participants to practice NBMT © without judgment, honestly, and focusing on one thing at a time.
Nature-Based Guided Meditation	Being Away Extent Soft Fascination Compatibility Reperceiving	1:05–1:20	Facilitator will prepare participants for the guided meditation and will guide a 10-min Nature-Based Guided Meditation specific to the natural environment of the group.
Reflective Journaling	Extent Soft Fascination Reperceiving	1:20–1:25	Participants will reflect on three prompts focused on Attention, Attitude, and Intention and write their responses.
Group Conclusion	Compatibility Reperceiving	1:25–1:30	Participants will write their top three takeaways from NBMT © and share one with the group.

## Data Availability

Not applicable.
